# Advances and applications of bone grafts in dentistry: A short review

**DOI:** 10.6026/973206300214687

**Published:** 2025-12-15

**Authors:** Monica Rajmalani, Fizzah Dadani, Alberto Iturbe, Jagbir Kaur, Ola Wedat Alla, Nelly Mariela

**Affiliations:** 1 Department of Dental Surgery, Bharati Vidyapeeth University, Dental College and Hospital, Pune, Maharashtra, India; 2 Department of Dental Surgery, Central University of Venezuela, Caracas, Capital District, Venezuela; 3 Department of Dental Surgery, Sri Guru Ram Das Institute of Dental Sciences and Research, Amritsar, Punjab, India; 4 Department of Dental Surgery, Elrazi University, Faculty of Dentistry, Al-Azhari, Khartoum, Sudan, North Africaa; 5 Department of Dental Surgery, Antonio Narino University, Bogota, Colombia, South America

**Keywords:** Bone Grafts, autografts, guided bone regeneration, vertical ridge augmentation, sticky bone

## Abstract

Bone grafts in dentistry, focusing on surgical techniques for bone repair and reconstruction is gaining momenture. There are 3 main types: autografts derived from the patient, allografts obtained from donors and synthetic grafts composed of artificial materials. Bone grafting is employed in treating fractures and bone defects and restoring structural integrity during orthopedic procedures. Thus, graft healing through integration with existing bone while also considering risks such as infection and rejection are needed for careful surgical planning.

## Background:

Bone grafts have been used in medicine for centuries and play a critical role in oral and maxillofacial surgery, periodontics and implant dentistry. The first recorded case was in 1782 when a bone graft from a dog was used to treat a cranial defect in a Russian man [1]. The number of dental implants being placed in clinical practice is on the rise, driven by greater patient awareness and evidence supporting their long-term success, with nearly a million implants placed each year. Consequently, the use of bone grafts in dentistry has also increased [2]. A bone graft is a living tissue that is transplanted into a bony defect, either alone or in combination with other materials, to promote bone healing and new bone formation [3, 4]. Bone substitutes are "synthetic inorganic or biologically organic combinations which can be inserted for the treatment of a bone defect" to achieve the same purpose [5].

## Indications for bone grafting in dental treatment:

The following are the major indications of bone grafting in dentistry [6, 7]:

## Alveolar ridge augmentation for implant placement:

Bone grafts are used to increase the height and width of the alveolar ridge, providing sufficient bone for the placement of dental implants.

## Sinus augmentation for placement of dental implants:

In the posterior maxilla, bone grafts are placed, as a lack of sufficient bone is a contraindication for implant placement.

## Socket Preservation and Reconstruction:

To minimize bone loss following tooth extraction, bone grafts are placed in the extraction sockets. This leads to healing with minimal bone loss and successful implant placement later.

## Periodontal Defects:

Furcation defects and intrabony defects are treated with bone grafts. GTR, also known as Guided Tissue Regeneration, employs a barrier membrane that excludes the epithelial and connective tissue from the root surface during the healing phase, allowing hard tissue formation. Craniofacial developmental conditions like cleft lip, cleft palate, hemifacial microsomia and craniosynostosis. Cancer patients who undergo removal of bone tissue may require bone grafts for treatment. Maxillo-Facial Trauma with excessive bone damage or loss of facial structure.

## Qualities of an ideal bone grafting materials:

An ideal bone graft should exhibit several key qualities essential for successful integration and functionality. First, it should possess osteogenic properties, meaning it can stimulate the formation of new bone through the action of osteoblasts contained within it. Additionally, the graft should be osteoconductive, providing a scaffold that facilitates the growth of new bone tissue [3, 8]. Furthermore, an effective bone graft should be osteoinductive, allowing new bone formation by activating the host mesenchymal cells [1]. These characteristics ensure that the graft not only supports the structural integrity of the bone but also enhances the healing process.

## Types of bone graft materials in clinical dentistry:

Autografts are obtained from the same individual in whom intraoral sites, such as the mandibular ramus, or extraoral sites, like the iliac crest, make favorable donor sites. Autografts can take several forms, including cancellous bone, cortical bone, vascularized cortical bone, bone marrow aspirate, autogenous tooth bone grafts and platelet-rich plasma (PRP). These grafts are significantly osteogenic, osteoinductive and osteoconductive in nature, making them the gold standard for many clinical applications. However, their use is constrained by factors such as donor site morbidity, prolonged clinical time and the limited availability of graft material [9]. Allografts are grafts obtained from the same species and processed to minimize immunogenicity. Human donor bone may be in the form of fresh-frozen, freeze-dried bone allografts (FDBA), or demineralized freeze-dried bone allografts (DFDBA). Although allografts do not contain living osteogenic cells, they can still support bone regeneration depending on the degree of processing and demineralization. They eliminate the need for a second surgical site but may pose risks such as disease transmission and variable resorption [10]. Xenografts are derived from non-human species, such as bovine or porcine, with deproteinized bovine bone being the most commonly used. They are meticulously processed to remove organic components, reducing immunogenicity. Xenografts serve primarily as osteoconductive scaffolds for osteoblastic activity and are widely used in socket preservation and sinus lift procedures. Their slow resorption rate and lack of osteoinductive potential may limit their effectiveness in certain regenerative procedures. Commercially available products include BioOss, OsteoGraf and CeraBone [11]. Alloplastic grafts are synthetic bone substitute materials generated to mimic the biological properties of natural bone. These materials include ceramics, such as hydroxyapatite (HA), tricalcium phosphate (TCP) and biphasic calcium phosphate, bioglass, or silicate-based ceramics; metals such as nickel-titanium; polymers such as polymethacrylate (PMMA) and polyglycosides; and cements such as calcium phosphate [12, 13].

## Recent advances in graft materials and addition of biologic enhancers:

Recent advancements in bone grafting include 3D-printed ceramic scaffolds that allow for precise defect adaptation and optimized porosity. These scaffolds are designed to mimic the mechanical environment of bone and enhance angiogenesis. Clinical trials have demonstrated promising outcomes in both horizontal and vertical bone augmentation [14]. Silicate-based ceramics, generally composed of silicon dioxide, sodium oxide, calcium oxide and phosphorus pentoxide, are enhanced with other minerals such as calcium, sodium, hydrogen and phosphorus. Latest improvements introduced more stable components, such as potassium oxide, magnesium oxide and boric oxide, which enhance bioactivity, promote osteogenesis and facilitate bone-biomaterial integration. Silicate-based ceramics, particularly bioactive glass 45S5, have gained attention for cleft grafting procedures. It promotes mineralization and supports soft tissue integration. In long-term clinical follow-up, this material has shown over 70% success rates in secondary alveolar cleft repairs [15]. Nanocomposites are another area of innovation, used to treat small bone defects that incorporate nano-sized fillers such as nano-hydroxyapatite into biodegradable matrices, improving osteoblast proliferation and scaffold strength. Though still under clinical evaluation, nanocomposites show potential in larger defects with tailored degradation profiles [16]. Hydroxyapatite closely resembles human bone in composition and structure. The creation of nano-sized hydroxyapatite (HANPs) using various synthesis methods helps to mimic natural bone. It is bioactive, biocompatible and anti-inflammatory and it promotes osseointegration and bone regeneration. Nano-HA graft materials enhance angiogenesis, leading to quicker bone healing and allowing early implant placement. Recently, implants have been coated with nano-HA particles to improve wettability and surface energy, which enhances protein and cell adhesion. In vivo studies show that these implants coated with hydroxyapatite nanoparticles better support osseointegration and new bone growth [17]. Tricalcium phosphate is available in two forms, α-TCP and β-TCP. β-TCP can be reabsorbed and replaced by native bone easily. It has mechanical properties similar to cancellous bone and is suitable for load-bearing applications. Its surface can be enhanced with polylactic-co-glycolic acid to boost mechanical strength and support bone growth. Being a promising alternative to autologous bone grafts, it provides healing rates and clinical outcomes similar to autografts, but with fewer complications [18]. Octacalcium phosphate is a calcium phosphate-based material with a calcium-to-phosphorus ratio of 1:33. Though thermodynamically unstable, it is a precursor to biological hydroxyapatite; it gets converted into HA in the human environment. Studies show that bone grafts containing OCP, combined with materials like collagen, gelatin and alginate, promote better bone regeneration than HA or TCP [19]. Growth factors and infused living osteogenic cell-based bone substitutes include Bone Morphogenetic Protein (BMP), Platelet-Derived Growth Factor (PDGF) and Insulin Growth Factor (IGF). Studies show that bone substitute bioactivated materials combined with growth factors, mesenchymal stem cells and multipotent cells from bone marrow enhance this graft healing process, especially in patients with bisphosphonate-related osteonecrosis of the jaw (BRONJ) [20]. BTE and regenerative medicine (RM) have developed a new concept of utilizing scaffolding nanosystems either alone or combined with growth factors and cell or gene delivery. This concept, termed tissue-engineered construct (TEC), may enhance bone repair and regeneration [21].

## Surgical techniques for ridge augmentation:

A series of adaptive changes leads to alterations in the alveolar process vertically and horizontally. Bone grafting procedures help to ensure a sufficient bone volume following tooth loss. Bone augmentation is justified in three circumstances: when an implant cannot be placed in its ideal position, its long-term success or primary stability is compromised, or high aesthetic requirements are demanded [22].

## Guided bone regeneration:

A well-established technique for augmenting deficient alveolar ridges, known as guided bone regeneration, fulfills the principles of primary wound closure to achieve aseptic healing and limit wound contraction. Corticotomy, a surgical procedure employing minimal penetration of the cortex or outer layer of bone, allows the migration of cells with angiogenic and osteogenic potential and the formation of new blood vessels. To guarantee the proliferation of the bone-forming cells, space creation and its maintenance are achieved by using barrier membranes. Resorbable membranes are prone to collapse, whereas non-resorbable membranes, particularly titanium-reinforced ones, are more effective. Ensuring the clot stability with a membrane helps bring cytokines, growth factors and signaling molecules that help to prevent fibrous encapsulation of the graft, which prevents graft failure [23]. The use of barrier membranes also fulfills the principle of compartmentalization, which creates a separated space, ensuring bone formation while acting as a passive barrier to preclude soft tissue ingrowth [24].

## Block grafting: Onlay, Inlay and cortical plates:

Onlay grafting is a traditional method from orthopedic and cranio-maxillofacial surgery that involves securely attaching a bone block directly onto the target site. This direct provision of bone-forming cells into the defect heals through an orchestration of cellular, vascular and architectural events, starting with exclusively providing nutrients to the bone block by plasmatic circulation. Capillaries penetrate the graft to form an osteoid, which completely revascularizes within 8 weeks. Autogenous bone block grafts have the main advantage of being fixed with osteosynthetic screws, as opposed to particulate bone matter [25]. The plate or the shell technique utilizes a thin cortical bone block to restore the contours of the alveolar ridge. After ridge augmentation, the implant placement, combined with relining with a particulate xenograft and a resorbable membrane, helps achieve low resorption rates [26]. The inlay bone block technique aims to restore the height of the bone crest by interposing the graft within a segmental osteotomy of a vertical defect. This helps the new bone to grow in a coronal direction, without detaching the periosteum, soft tissues and related vascular network [27].

## Distraction osteogenesis:

An advanced hard and soft tissue engineering protocol for the treatment of severe bone deformities such as cranio-maxillofacial malformations, orthognathic surgery and the correction of atrophies. The principle of distraction osteogenesis depends on the segmentation of the atrophic bone and the progressive displacement of the bone and the soft tissues coronally. This creates a secluded chamber for the formation of new bone and soft tissues. The bone is displaced by a segmental osteotomy that separates the atrophic crest from its basal bone. Then, the osteotomy line is opened using a distraction device, applying slow and calibrated tension forces. The graft chamber undergoes an intramembranous ossification process in a centripetal direction, starting at the periphery of the graft [25].

## Surgical techniques for maxillary sinus lift:

Sinus floor elevation can be achieved using a lateral Antrostomy procedure or the Osteotome technique with a crestal approach. The lateral approach for sinus augmentation involves making an incision above the mucogingival junction, followed by the elevation of a mucoperiosteal flap. The osteotomy is performed about 4-5 mm above the maxillary sinus floor, where a rectangular window is created in the canine fossa, allowing for sufficient access for dissection and sinus membrane elevation without its perforation. The crestal approach employs an incision made palatal to the alveolar crest, followed by vertical releasing incisions, allowing for reflection of the mucoperiosteal flap while avoiding perforation. Antrostomy is performed using a speed reduction gear handpiece with appropriate irrigation. Once the palatal osseous lid is removed, the sinus membrane is delicately lifted and the sinus cavity is filled with the graft. Finally, the excess graft material is cleaned and the flaps are repositioned to achieve primary closure [28].

##  Complications associated with bone grafts:

Wound dehiscence, the most common complication, occurs when the graft or membrane is exposed to tension in the surgical flap, resulting in poor vascularization or infection. To ensure the success of a bone graft, the bone material must be placed gently and not overpacked, allowing for the formation of blood vessels that bring in the proteins and growth factors required for healing. Improper placement of the membrane, especially lacking sufficient soft tissue coverage, increases the risk of membranes being exposed and infected. Suppose there is a tension in the flap. In that case, there is a risk of loss of keratinized tissue, mostly on the buccal side, which is avoided by providing up to eight weeks of healing time after tooth extraction prior to placing the gum graft to allow for the natural formation of soft tissues, resulting in proper healing without tissue loss [29]. Infection poses a major challenge, resulting from contamination during surgery or secondary healing, where various contributing factors, such as smoking, immunosuppressive conditions and a history of periodontal disease, can significantly contribute to infection. Once these conditions are recognized early, one can improve patient outcomes and increase the chance of a successful graft [30]. Graft Resorption, particularly seen in vertical bone augmentation, occurs with inadequate stabilization of the graft or obstructed vascular supply to the graft, often observed in autologous grafting procedures, where bone is harvested from the patient's own body. Over a period of time, these may lead to loss of bone volume and may require additional grafting [29]. Iliac bone grafts are observed to have experienced resorption over the years, regardless of initial stability [31]. Donor site complications are observed at the sites where bone graft material is extracted from. It is most commonly seen during gathering tissue from the symphyseal region of the mandible, while the ramus donor site also presents notable issues and may be presented as neurosensory disturbances, especially in the anterior region. Post-surgical neuropraxia is typically transient but may persist in some cases, particularly when the mental nerve is inadvertently traumatized [32].

## Evidence-based outcomes and long-term success in implant dentistry:

Selecting the correct material for a successful bone graft requires a thorough review of oral conditions and patient preference. Based on a detailed review of different factors, autologous block grafts (taken from the patient's own body), composite grafts (a mix of materials) and blood-derived products are currently the preferred choices for most bone grafting procedures. After an average of 3 to 5 years of follow-up, block grafts have shown good results, with high implant success and survival rates [33]. While grafts from human or animal sources are more expensive, they preserve bone better than synthetic materials. However, studies show that both allografts and xenografts give similar results clinically [34]. With continuous technological advancements, natural bone grafts have gradually been replaced by synthetic bone substitutes. Recently, the development of hybrid grafts and bone substitutes, which release bone morphogenic proteins or platelet-derived growth factors in a controlled manner, utilizing growth factors and living osteogenic cells, is gaining popularity. However, more work is needed to develop dental biomaterials that have a porous structure, mechanical stability, controlled degradation and remodeling ability, which is comparable with the rate of new bone formation [7]. "Sticky bone," a special material used to help bone heal and grow, is made by mixing small pieces of bone with the patient's own blood parts that help healing, like PRF and growth factors. This mix stick well to the area, help the bone and nearby tissues grow faster, slowly releases healing substances and stops soft tissue from growing into the bone. Based on current evidence, sticky bone yields outstanding clinical and radiographic results across diverse periodontal surgeries and has proven effective in addressing furcation defects, infra-bony defects, dehiscence defects, ridge augmentation and alveolar ridge preservation procedures. Notable outcomes include favorable vertical and horizontal bone gain; clinical attachment improvement, reduced periodontal probing depth and enhanced radiographic bone fill [35]. Therapeutic outcomes of bone grafting are significantly impacted by the extraction sites, demonstrating significant anatomical variability, including phenotypic characteristics such as keratinized tissue width, gingival thickness and bone volume and integrity, which can significantly impact therapeutic outcomes. Additionally, systemic and patient-related factors may influence the healing process. Respecting patient autonomy is an important factor to consider, as patients often prefer certain types of grafts while declining others. Allografts and xenografts elicited the highest refusal rates among patients, while autologous and alloplastic bone grafts were the most accepted types [36].

## Conclusion:

Bone grafting continues to play a vital role in dentistry, offering diverse options from autografts to advanced synthetic substitutes. Recent innovations, including nanocomposites, bioactive glass and biologically enhanced scaffolds, have improved regenerative outcomes while reducing limitations of traditional grafts. Careful material selection, surgical precision and patient-centered planning remain essential for predictable long-term success.
